# More Than the Win: The Relation between Appetitive Competition Motivation, Socialization, and Gender Role Orientation in Women's Football

**DOI:** 10.3389/fpsyg.2017.00547

**Published:** 2017-04-13

**Authors:** Danie Meyer-Parlapanis, Sabrina Siefert, Roland Weierstall

**Affiliations:** ^1^Psychology, University of KonstanzKonstanz, Germany; ^2^Department of Psychology, Medical School HamburgHamburg, Germany

**Keywords:** appetitive competition motivation, socialization, gender role orientation, upbringing style, women's football, motivation, gender

## Abstract

The ability to produce peak performance plays a decisive role in the success of athletes in competitive contest situations. Levels of appetitive competition motivation (ACM), i.e., the desire to defeat an opponent independent of secondary reinforcing factors, were assessed in professional female football/soccer players in the premier and regional leagues, using club level as the measurement of sport success. Furthermore, the influence of social environments predominantly encouraging masculine and competitive play behavior and the players' perceptions of their own gender role orientations were investigated. Ninety female football players from the German premier league (44) and regional leagues (46) participated (age: *M* = 24, *SD* = 5 years). Questionnaires ascertaining ACM and self-perceptions of gender via gender-role stereotypes, childhood play behavior and style of upbringing were utilized. Premier league athletes showed a significantly greater inclination toward direct sporting confrontations. Almost 50% of the variance in ACM between the premier and regional league athletes was determined by modern upbringing style and the development of gender roles not corresponding to classic female gender stereotypes. The results emphasize the significance of ACM as an important facet in competitive sports and illustrate the influence of socialization on athletic performance.

## Introduction

The factors yielding peak performance in competitive sports have consistently been a focal point of sports psychology. Motivation, identified as one such factor, has eluded a commonly agreed upon definition (Ford, 1992) due to its complex, multifactorial interactions between biological, psychological, environmental and social dimensions (Hareli and Weiner, 2002). In line with this multidimensional understanding of motivation and the general concession that behavioral aims and their corresponding cognitions and affects drive commitment and performance levels (Gould et al., 2002), the following study examined the impact of *appetitive competition motivation* (ACM), i.e., the positive valence processing of direct competition with an opponent, on sports performance as well as its relation to social developmental factors. This was carried out by means of a survey of female football players from the premier league and regional leagues that examined social influences and gender role orientation.

### Motivation in the sporting context

The overarching framework of motivation utilized in this study is separated into intrinsic and extrinsic forms of motivation and how those forms interact with one another. The intrinsic form involves the motivation to know, to accomplish things and to experience stimulation (see Vallerand et al., 1989, 1992). In this study, we evaluate the desire to experience stimulation element as expressed in ACM in the premier and regional league athletes and then investigate its interaction with extrinsic motivators related to upbringing style and gender socialization.

Extrinsic motivation has been broken down into four forms that exist on a self-determination continuum, ranging from high (integrated regulations, i.e., motivation stemming from external values an individual has internalized as their own), medium high (identified regulation, i.e., motivation stemming from external values an individual identifies as important), medium low (introjected regulation, i.e., motivation stemming from values to which an individual simply adheres) and low (external regulation; i.e., motivation based on rewards) (see Deci and Ryan, 1985; Ryan and Deci, 2000). The current study assessed to which extent upbringing style and gender socialization functioned as extrinsic motivators in female athletes engaged in high-level football teams. External regulators, such as financial compensation, fame and increased social status etc., arguably strong extrinsic motivators especially when comparing premier league and regional league athletes, were not included in the present study due to questionnaire length constraints.

### Appetitive aggression and appetitive competition motivation

The present study has its origins in research on appetitive aggression in the area of clinical psychology (Elbert et al., 2010). Working with several thousands of combatants from former crisis areas, this research demonstrated that a majority of individuals involved in long-term combat developed a fascination with and enthusiasm for direct confrontation with the opponent independent of overriding aims such as resource acquisition, dominance, or the causes of the conflict. The act of hunting and defeating the opponent generates an intrinsic motivation that is associated with positive valence and can override self-control and inhibition mechanisms. This phenomenon contains a decisively functional component: combatants who process violence and confrontation with stressful or even traumatic events in an appetitive manner exhibit resilience to post-traumatic stress disorders. This resilience stems from the experience that, at the time of and even in retrospect of the confrontation, extreme wartime experiences were perceived as less stressful, disturbing and traumatic (Weierstall et al., 2011).

Aggression research in a clinical setting overlaps with investigations on motivation in a sport context. Sporting contest, with an interest in direct competition and the aim of defeating the opponent in the sport context, has already been described as analogous to our aforementioned research findings (Gill, 1993). Comparable characteristics were revealed: in studies on the emotional experience of the most successful moments in sports, athletes often report that they felt strong, sure of victory or (symbolically) like “a tiger” (Robazza et al., 2004; Ruiz and Hanin, 2004a). Activity in a sport context can also result in an experience of “flow,” in which the athlete completely blocks out external factors in the contest (Kowal and Fortier, 1999; Jackson and Eklund, 2002). Above all, group cohesion in the team sport context (Kleinknecht et al., 2014) operates analogously to the populations investigated. The desire to defeat the opponent can even cause athletes to ignore fair or sanctioned play and demonstrate aggressive tendencies (Kavussanu and Ntoumanis, 2003; Ryska, 2003). The fewer thoughts experienced by the players during the competition, the higher the associated level of performance (Williams and Krane, 2001).

The construct of ACM is grounded in a combination of social-cognitive and emotionally focused theoretical frameworks. First, the social-cognitive perspective (see Duda, 2001), focuses on an athlete's subjective evaluation of a situation and their perceived probability of achieving a particular aim given the circumstances and available resources. The studies—some in the context of football (Van-Yperen and Duda, 1999; Miller et al., 2004)—demonstrated that the pursuit of success and superiority in competition generates motivation for sports performance (Elliot and Conroy, 2005). Secondly, emotionally focused approaches distinguish between approach aims (positive valence) and avoidance aims (negative valence) (Elliot, 1999). They often focus on the “optimal” level of arousal. Performance ability is dependent on an appropriate level of arousal, and this arousal, in connection with the competition, can lead directly to the mobilization of physical resources and strengthen concentration and commitment. Negative states, such as fear of defeat (Bray et al., 2000), and positive states, such as victory in competition (Lane et al., 1995; Cerin et al., 2000), have activating effects. Over- and under- stimulation alike can cause a performance capacity decline, for example when concern about defeat turns into acute fear (Ruiz and Hanin, 2004a,b) or if concentration wanes as a result of insufficient challenge (Fredrickson and Branigan, 2005; McCarthy, 2011). Both social-cognitive and emotionally focused approaches suggest that victory or the anticipation of victory in competition represents a significant motivational factor. However, to our knowledge there has been no systematic research to date which investigates the positive valence processing and motivational significance of high contact, direct opponent confrontation in a sport context and examines whether motivation is solely determined by the competition itself.

Therefore, when a phenomenon analogous to appetitive aggression exists in the sports context, sports psychology research stands to benefit not only from the associated positive valence intrinsic motivation but also from the identification and prevention of mental health and behavioral complications potentially produced in the context of competition. Hereby, ACM is investigated in the context of professional female football players. The term motivation was selected because, while unsanctioned aggressive behavior also occurs in sport, there is no direct connection to the classical definitions of aggression that involve an intention to cause harm (Anderson and Bushman, 2002).

### Sports, socialization and gender role orientation

In the context of sports, socialization factors such as the sports environment and leaders such as coaches or trainers have emerged as significant influencing factors for the development of performance (Bloom et al., 2003; Côté and Sedgwick, 2003). Furthermore, studies on children and adolescents indicate that play behavior, and thereby peer interaction, has a predominant influence on performance in a sporting context, highlighting manners in which socializing influences extend far beyond the club setting (Côté et al., 2003; Griffin and Butler, 2005; Ericsson, 2007). In investigations on gender effects in sport contexts, however, there is often a continued reliance on outdated research findings that exclusively represent biological or genetic determinism and neglect such socialization influences (Gildemeister, 2008; Nestvogel, 2008; Reidy et al., 2009). Despite the widely held perception that the physical performance of females often does not match that of males in sports (Åstrand et al., 2003), recent studies nonetheless indicate that sex differences become less significant under consistent training conditions (Hodges et al., 2004). A particularly decisive variable here is access to training opportunities (Musch and Grondin, 2001) and the encouragement of behavior and characteristics associated with strong sports performance. We therefore postulate that, just as socialization influences sport performance even outside the sport context, stereotypical gender roles (cf. e.g., Richardson and Hammock, 2007) can also have an influence on sport performance. This presumably has particular significance for women's football, especially in light of the ban on women's football in Germany until 31st October 1970. Femininity is stereotypically understood to consist of expressive qualities such as caring, selflessness, emotionality and empathy (cf. Abele, 2003), each of which seemingly contradict the assertive, competitive orientation considered to be integral in the context of football. Moreover, since the beginning of the nineteenth century century, the opinion has persisted that women who are active in this type of sport context acquire typically masculine characteristics and thereby upset the natural hierarchy of society (Eagly and Koenig, 2006). As female athletes in male domains are often confronted with the label of androgyny and are subjected to sexual discrimination (Krane, 2001), socialization effects likely play a vital role in the evaluation of performance level and commitment in women's football.

### Objectives

Using club level as the measurement of sport success, ACM levels in female football players in the premier and regional leagues were assessed. Furthermore, we investigated the influence of social environments predominantly encouraging masculine and competitive play behavior and the players' perceptions of their own gender role orientations.

## Method

### Sample

The participants were recruited by letters sent to 12 premier league and 17 regional league clubs. Out of these, 4 premier league clubs and 12 regional league clubs agreed to participate. One premier league club declined to participate. The other clubs did not reply. The club names will not be published in accordance with the promised anonymity of the survey and at the request of the participating clubs. The study was implemented using a questionnaire format with an a-priori sample size calculation to detect differences across the two leagues and the sampling was concluded a sufficient number of questionnaires had been received. In a first period of data collection, questionnaires were sent to the clubs together with general information, declarations of consent and procedural notes on filling out the questionnaires. The completed questionnaires were collected together with the declarations of consent. The questionnaires were collected separately in unmarked envelopes in order to guarantee the players' anonymity. In a second period of data collection, to increase user-friendliness and improve confidentiality, an online version of the questionnaire was created using “online survey” software (umfrageonline.com) and an email was sent to the participants with a link to the survey. The processing time was between 20 and 30 min.

Out of a total of 90 participants (age: *M* = 24, *SD* = 5 years), 44 played in the premier league and 46 in the regional league. As shown in Table 1, the two groups do not differ in any football-specific or potentially confounding variables.

**Table 1:** **Demographic data for premier and regional league players**.

**Variable**	**Premier league players**	**Regional league players**	**Test statistic**
Age	*M* = 23, *SD* = 4	*M* = 25, *SD* = 5	*t*_88_ = 1.46, *p* = 0.147
**PLAYER POSITION [NUMBER (%)]**			
Goal	5 (11)	1 (2)	Chi^2^ = 6.74, *p* = 0.081
Defense	13 (30)	23 (50)	
Midfield	21 (48)	15 (33)	
Attack	5 (11)	7 (15)	
Number of games played in the last season	*M* = 19, *SD* = 7	*M* = 16, *SD* = 9	*t*_88_ = 1.77, *p* = 0.079
Average playing time in the last season (min.)	*M* = 72, *SD* = 29	*M* = 73, *SD* = 29	*t*_88_ = 0.19, *p* = 0.848

All participants were informed of the aims of the study and gave their written consent to participate. Participation in the study was voluntary, and there was no financial compensation. The Ethical Review Board at the University of Konstanz approved this study.

### Design

A quasi-experimental design was selected with the group factor *Club Level* to investigate group differences between players from the premier and regional leagues. The Club Level variable was coded as “1” (premier league players) and “0” (regional league players) and was also used as a dummy variable in the evaluation. Dependent variables used were the variables *Appetitive Competition Motivation, Masculine Socialization, Masculine Role Model*, and *Modern Upbringing*.

### Material

#### Questionnaire to ascertain appetitive competition motivation

An adapted version of the Appetitive Aggression Scale (AAS, Weierstall and Elbert, 2011) was used for the questionnaire to ascertain ACM. The AAS, comprising 15 items, is available as semi-structured clinical interview as well as a self-rating tool to measure appetitive aggression and, to date, has been successfully validated with several thousand participants with different levels of aggression. It demonstrates both a satisfactory factorial and criterion validity and sufficient psychometric features (including Cronbach's Alpha = 0.85). Proven to be a valid and reliable questionnaire, it has already been implemented in its original form with female combatants and civilians (e.g., Augsburger et al., 2015; Meyer-Parlapanis et al., 2016) and as a modified self-assessment instrument in civilian populations (e.g., Weierstall et al., 2014). In the current study, the existing items on the AAS were reformulated and adapted to the competitive elements in the sporting context (for example: original item: “Does the challenge of defeating a strong opponent make the fight more pleasurable for you in comparison to the defeat of a weak opponent?” → competitive item: “Does the challenge of winning against a particularly strong team make the game more exciting in comparison with winning against a weak team?”; original item: “When you fight, do you stop caring about whether you could be killed?” → competitive item: “During the game, do you fear that you might be injured or at least give this some thought?”). The item on sexual arousal related to competition from the combatant version was not included. The answers to each item were coded with the values “0” to “4” on a 5-point Likert scale, with high values indicating a strong competitive motivation. To make it easier to answer the items and to increase the validity of the answers, descriptions of the respective response levels for the items were given, which were based on the AAS manual. The variable *Appetitive Competition Motivation* was calculated for each person by calculating the average item score. Cronbach's Alpha gave a value of 0.87. A principal axis factoring analysis was calculated to determine the factorial validity and revealed that all the items loaded onto a main factor, accounting for 38% of the questionnaire's variance.

#### Masculine socialization

Visual analog scales (VAS), when carefully constructed, have proven effective at representing subjective assessments (Wewers and Lowe, 1990). Thereby, for the remaining three variables, 10 cm-long VAS were employed with verbal anchors at the poles (values between 0 and 100). Although questionnaires such as the Bem Sex Role Inventory (Bem, 1981) exist for the construct of gender role orientation, VAS were used for reasons of efficiency and practicality. The variable *Masculine Socialization* was operationalized by “boyish activities and toys in childhood and adolescence” and ascertained according to the participants' self-perception. The poles were labeled with the anchors *not at all* and *very*. A high value on this scale therefore indicated a tendency to perceive one's socialization as masculine.

#### Modern upbringing

To ascertain a modern upbringing, a “traditional or modern upbringing” scale was provided and labeled with the anchors *traditional* and *modern*. In order to validate self-perception with regard to measurement of the underlying construct, the participants were also asked to provide adjectives they associated with the two terms. The qualitative evaluation of the adjectives revealed that the evaluation of stereotypically traditional and modern upbringings corresponded to the attributes of descriptions given in the relevant psychological literature (cf. introduction) (for example: traditional: “home-loving,” “familial,” “conservative,” or “classical role allocation”; modern: “cosmopolitan,”, “tolerant,” “liberal,” or “daring”). High values for these variables indicate a predominantly modern upbringing.

#### Masculine role orientation

To capture the participants' self-perception of their own role orientations, they were asked whether they perceived themselves as orientated toward a more feminine or masculine role. The attributes *feminine* and *masculine* were set as anchors. High values on this scale indicate a tendency toward a masculine role orientation. Qualitative interviews were able to validate the association of a stereotypically masculine role model with adjectives such as “performance-orientated,” “assertive,” and “independent.”

#### Implementation

The study was implemented using a questionnaire format. In a first period of data collection, questionnaires were sent to the clubs together with general information, declarations of consent and procedural notes on filling out the questionnaires. The completed questionnaires were collected together with the declarations of consent, which were filled out separately in order to guarantee the players' anonymity. In a second period of data collection, to increase user-friendliness, an online version of the questionnaire was created using “online survey” software (umfrageonline.com) and an E-mail was sent to the participants with a link to the survey. The processing time was between 20 and 30 min.

#### Data evaluation

The data was evaluated using the program R-Statistics, Version 3.1.1 for Mac OS X 10.9. The significance level was set at Alpha = 5%. Effect sizes were calculated using the program g^*^ power 3.1 (Faul et al., 2007).

## Results

### Group differences between players in the premier and regional leagues

Table 2 shows the intercorrelation matrix for the four main dependent variables, shown separately for the premier league and the regional league players. The first step was to determine group differences in the dependent variables between premier league players and regional league players. With the exception of the masculine role model (*M*_premier league_ = 62, *SD*_premier league_ = 24, *M*_regional league_ = 63, *SD*_regional league_ = 26, *t*_88_ = 0.16, *p* = 0.871, *d* = 0.04), there were significant group differences in the three remaining variables: players in the premier league not only reported a more modern upbringing (*M*_premier league_ = 59, *SD*_premier league_ = 23, *M*_regional league_ = 44, *SD*_regional league_ = 23, *t*_88_ = 2.99, *p* = 0.004, *d* = 0.66) and a more masculine socialization (*M*_premier league_ = 79, *SD*_premier league_ = 13, *M*_regional league_ = 60, *SD*_regional league_ = 26, *t*_68.847_ = 4.387, *p* < 0.001, *d* = 1.30), but above all the premier league players exhibited higher values for ACM (*M*_premier league_ = 2.45, *SD*_premier league_ = 0.56, *M*_regional league_ = 1.72, *SD*_regional league_ = 0.47, *t*_88_ = 6.70, *p* < 0.001, *d* = 1.41). The large effect sizes indicated large differences in the dependent variables.

**Table 2:** **Intercorrelation matrix of the central dependent variables**.

**Club level**	**Variable**	**Masculine socialization**	**Masculine role model**	**Appetitive competition motivation**
Regional league	Modern upbringing	*r* = 0.27, *p* = 0.069	*r* = 0.06, *p* = 0.683	*r* = 0.27, *p* = 0.071
	Masculine socialization		***r* = 0.58, *p* < 0.001**	*r* = 0.12, *p* = 0.414
	Masculine role model			*r* = −0.03, *p* = 0.865
Premier league	Modern upbringing	*r* = 0.07, *p* = 0.672	*r* = 0.25, *p* = 0.110	***r* = 0.51, *p* < 0.001**
	Masculine socialization		***r* = 0.46, *p* = 0.002**	***r* = 0.47, *p* = 0.001**
	Masculine role model			***r* = 0.51, *p* < 0.001**

*Significant coefficients are in bold*.

### Prediction of appetitive competition motivation

The second step was to investigate the influences of Masculine Socialization, Modern Upbringing and Masculine Role Model on ACM on the basis of a multiple linear regression analysis. As no significant deviation from the normal distribution was ascertained for the ACM variable (Kolmogorov Smirnov Test *p* = 0.340), no further distribution assumptions had to be taken into account. The variables Masculine Socialization, Modern Upbringing, Masculine Role Model, the dummy variable Club Level and all the possible double interactions were included in the complete model. The final model was selected according to the Akaike Information Criterion (AIC, Akaike, 1987). In order to reduce multicollinearity, the variables were centered to the average value before forming interactions (cf. Kleinbaum et al., 1998).

**Figure 1:**
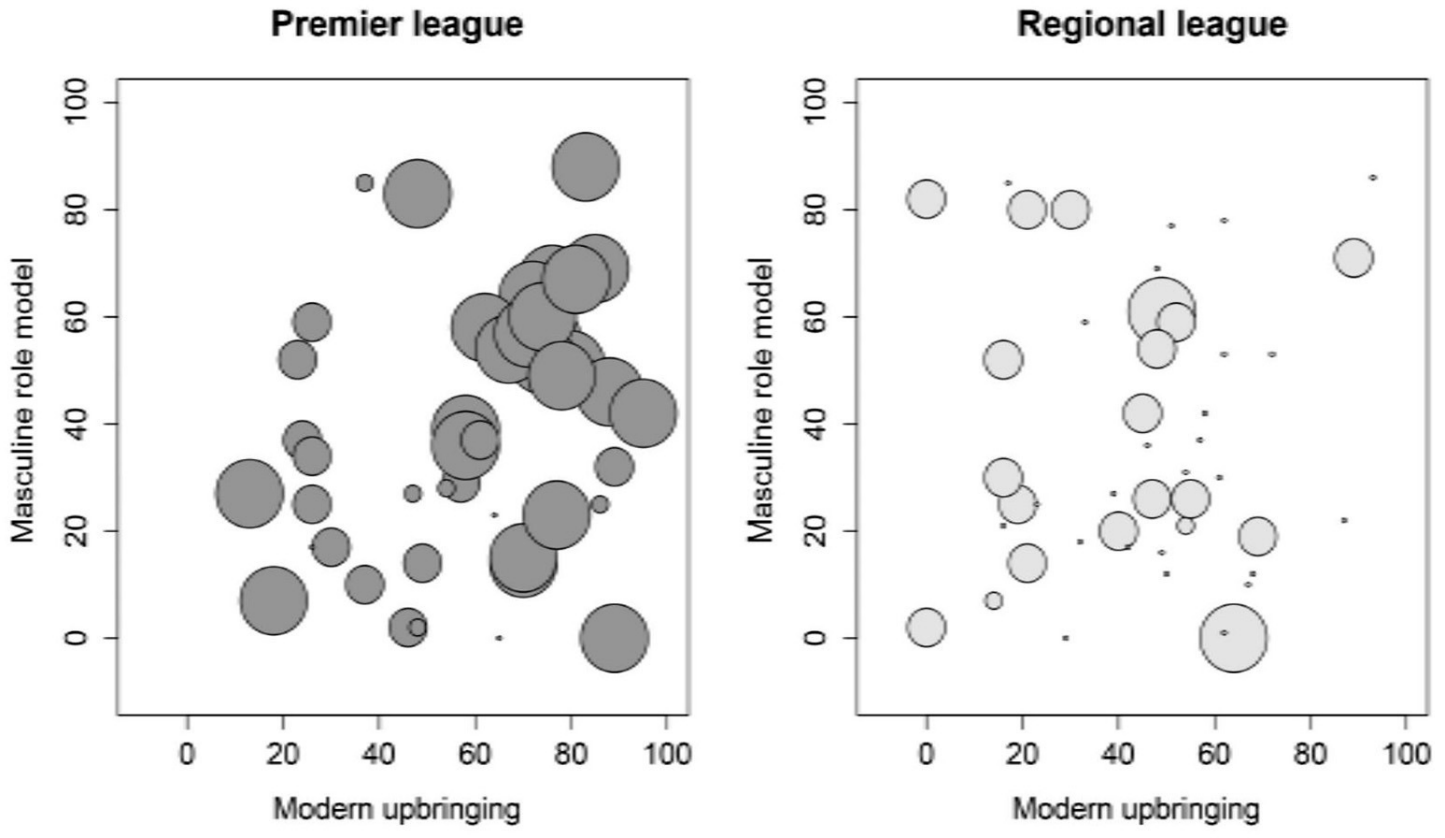
**This diagram shows the relationship between the following variables: masculine role model, modern upbringing and appetitive competition motivation, with separate diagrams for premier and regional league players**. The level of appetitive competition motivation is represented by the size of the bubbles, whereby raw values for appetitive competition motivation for the entire group were categorized into four quartiles. High values in the other two variables indicate a more modern upbringing or a tendency toward a more masculine role model.

The model that was most suitable for predicting ACM according to the AIC included the variables Masculine Socialization, Modern Upbringing, Masculine Role Model and the two interaction terms between Club Level and Modern Upbringing and Masculine Role Model (Table 3). This model, which was able to account for over half of the total variance ($R_{\text{adj}}^{2}$ = 0.52), demonstrated not only a large effect size [*F*_(6, 83)_ = 17.318, *p* < 0.001, *f* ^2^ = 1.25] but also sufficient statistical power [(1 − β) > 0.99]. While socialization conditions had a significant influence on both groups insofar as a greater encouragement of stereotypically masculine activities was associated positively with the development of ACM, the other two variables only played a significant role in the group of premier league players. Both a modern upbringing and a tendency toward a masculine role model were associated with ACM in premier league players but not in regional league players. This connection is illustrated in Figure 1. In the bubble diagram, the four different bubble sizes represent the quartiles in which the raw values for competitive motivation of the individual players lie in relation to the total group. As described above, competitive motivation values in the upper two quartiles are not only predominantly found in the group of premier league players; they are also predominantly clustered in the area of the premier league players who reported a modern upbringing and perception of a masculine role model. The residual diagnosis revealed no indications of either multicollinearity (maximum VIF value 1.87) or rogue results (maximum value for Cook's *d* = 0.14). A comparison of the residual variances between the two groups also produced no indication of statistically significant differences (Levene Test, *p* = 0.529). The model used therefore not only fulfilled all the requirements for the merit of regression models but also indicated robust results.

**Table 3:** **Regression model to predict appetitive competition motivation**.

	**Appetitive competition motivation**	
	**β**	***p***
Masculine socialization	**0.26**	**0.011**
Masculine role model	−0.08	**0.340**
Modern upbringing	0.05	0.551
Club level	**0.45**	**<0.001**
Modern upbringing × Club level	**0.32**	**<0.001**
Masculine role model × Club level	**0.22**	**0.006**
$R_{\text{adj}}^{2}$	**0.52**	**<0.001**

*Standardized regression coefficients are shown here. The regression model was selected according to the Akaike Information Criterion (AIC). Significant coefficients are in bold*.

The final step was to calculate two separate linear regression analyses, one for each group, which predicted ACM by means of the other three predictor variables in order to predict the amount of variance accounted for in each group. Of the 52.4% variance accounted for in the total model, only 6% was attributable to effects in the group of regional league players [*F*_(3, 42)_ = 1.96, *p* = 0.135, *f* ^2^ = 0.14], while the remaining 46% of variance was explained by effects in the group of premier league players [*F*_(3, 40)_ = 13.34, *p* < 0.001, *f* ^2^ = 1.11]. No influence of playing position on the level of ACM was ascertained [*F*_(3, 86)_ = 2.15, *p* = 0.097, η^2^ = 0.07].

## Discussion

The present study reveals a high degree of ACM among a majority of the athletes, which verifies that the competitive contest against an opponent in and of itself can be experienced as appetitive and positively reinforcing. This encompasses not only game preparation and fantasies about “crushing the opponent into the ground,” but also flow experiences during the game and potentially leads to less controlled play involving deliberate fouls to intimidate or humiliate the opponent and to less concern for personal injury. This form of ACM, which, borrowing from the original clinical research, can also be described as “fighting spirit,” seems therefore to be a significant intrinsic attraction goal during the contest. If the club level is taken as a measurement of sporting success, the more successful athletes exhibit greater fighting spirit and also greater pleasure in the football contest. When, analogous to the clinical research, this form of positive valence processing of sporting contests also causes athletes in an appetitive and victory-orientated mode to be less susceptible to distractions, fears and stressors during the contest (cf. Weierstall et al., 2011), targeted encouragement of ACM could considerably increase performance levels in the sporting context. Furthermore, it would be advisable to classify this in the overall context of motivational research by integrating this facet into existing social-cognitive or emotionally focused approaches.

A further significant result of this study is the connection between ACM and socialization factors. A motivational context that encourages a high level of self-efficacy and the belief that one is able to successfully manage situations are elements conducive to the ability to produce peak performance (Ntoumanis and Biddle, 1999; Parish and Treasure, 2003). Above all, frustration and stress often cause players to withdraw from competitive sports (Kleinert and Raven, 2011). The present study, however, demonstrates that the development of self-efficacy is formed far beyond the sporting context alone and that considerably greater attention should be paid to biographical and life history factors. It is also the case that professional athletes demonstrate a high level of performance commitment in the pursuit of goals even outside the sporting context (Durand-Bush and Salmela, 2002; Bull et al., 2005). This study therefore on the one hand emphasizes that success in sports is not only developed at a club level but is also closely linked with an individual's more permanent characteristics and is therefore subjected to their biographical and everyday experiences. The use of biographical elements, including in particular the processing of aversive and distressing experiences, could therefore not only provide a beneficial point of contact between clinical and sports psychology but could also lead to an enhancement of the athletes' performance in a manner that has so far been largely neglected (Hanin, 2007). On the other hand, this study underlines the significance of socialization in the development of competitively orientated characteristics (Fine, 2010). In a socialization context that is more modern, more liberal and less strongly orientated toward classically stereotypical role models, female athletes seem to develop the same competitively oriented behaviors as male athletes (cf. Sobiech, 2006; Niederbacher and Zimmermann, 2011). This corroborates research emphasizing the social gender role as a counterweight to birth gender assignments in the explanation of gender effects (Koch, 2004; Keeler, 2007).

The perception of a stereotypically masculine role orientation in female athletes having a greater competitive orientation, directed toward assertiveness and strength, aligns with existing research findings (Lenzi et al., 1997; Archer, 2004). As other studies have shown that the masculinization of assertive female athletes can often be accompanied by acute psychological stress (Krane, 2001), the results of this study highlight the need for further research to pursue and update currently outdated and stereotyped standards and concepts.

This investigation was designed to investigate individual athletes participating in team sports. The clinical origins of ACM, appetitive aggression, were assessed in individuals having participated in military cohorts and the questions focused on acts of aggression that often were implemented in group settings (Weierstall and Elbert, 2011), while ACM, by extension, similarly focuses on individual athletes participating in a team sport setting with 4 of the 14 questions specifically referring to either teammates or the other team. The results of this study are not intended for application to athletes performing in individual sports.

## Conclusion

Female athletes performing in a sport context that has been both legally and socially viewed as a domain reserved for males preserve against numerous stereotypes and obstacles. This study highlights ACM as one of a myriad of influencers playing a role amidst a highly intersectional web of socializing factors related to family systems, gender identity and role modeling. Due to this complexity, further interdisciplinary research, including behavioral and biological markers, is needed to validate our current findings and deepen our understanding of the psychological underpinnings of individual development and functioning, both in sports and beyond, amidst varying exposure to social constructs and expectations.

## Ethics statement

This study was carried out in accordance with the recommendations of the Ethical Review Board at the University of Konstanz with written informed consent from all subjects. All subjects gave written informed consent in accordance with the Declaration of Helsinki. The protocol was approved by the Ethical Review Board at the University of Konstanz.

## Author contributions

DMP and RW oversaw project design, implementation and analyses, and co-wrote the manuscript. SS designed and implemented the project and conducted the analyses.

## Funding

This project was funded by the University of Konstanz.

### Conflict of interest statement

The authors declare that the research was conducted in the absence of any commercial or financial relationships that could be construed as a potential conflict of interest.
